# Depressive symptoms and health-related quality of life following acute myocardial infarction

**DOI:** 10.3389/fcvm.2026.1790367

**Published:** 2026-06-29

**Authors:** Manuel Mallol-Simmonds, Claudia Gay Rojas, Ivan Cañete Palta, Criss Diaz, Alfredo Parra-Lucares

**Affiliations:** 1 Cardiovascular Research Unit, Facutad de Medicina, Universidad de Chile, Hospital Clinico de la Universidad de Chile Jose Joaquin Aguirre, Santiago, Chile; 2 Hospital del Salvador, Santiago, Chile; 3 Cardiovascular Department, Hospital Clínico Universidad de Chile, Universidad de Chile Facultad de Medicina, Santiago, Chile; 4 Complejo Asistencial Dr Victor Rios Ruiz Los Angeles, Los Ángeles, Chile; 5 Universidad de Chile Facultad de Ciencias Quimicas y Farmaceuticas, Santiago, Chile

**Keywords:** acute myocardial infarction, depression, health-related quality of life, public health, SF-36

## Abstract

**Background:**

Depression and impaired health-related quality of life are common after acute myocardial infarction (AMI) and have been associated with worse clinical and functional outcomes. However, local data describing these dimensions during the post-infarction period in public healthcare settings remain limited.

**Methods:**

We conducted a cross-sectional observational study using data from the Prospective Myocardial Infarction Registry (REPRIM), including patients with ST-segment elevation myocardial infarction (STEMI) treated in a public tertiary care setting in Chile between 2019 and 2020. Health-related quality of life was assessed 30 days after the index event using the SF-36 questionnaire, and depressive symptoms were evaluated with the 17-item Hamilton Depression Rating Scale (HAM-D). Associations between quality of life and depressive symptoms were explored using multivariable linear regression models adjusted for age, sex, diabetes mellitus, hypertension and left ventricular ejection fraction, and mortality was described during the available follow-up.

**Results:**

During the study period, 121 patients with acute myocardial infarction were identified. Of the 100 patients included in REPRIM, 68 met the criteria for this subanalysis (alive at 30 days, successful contact and complete questionnaire data). Most patients exhibited at least mild depressive symptoms (94.1%), with a median HAM-D score of 12 (interquartile range 10–15). Higher depressive symptom severity was significantly associated with lower physical (PCS) and mental (MCS) component scores of the SF-36 in unadjusted analyses (*ρ* = −0.46 and *ρ* = −0.58, respectively; *p* < 0.001) and remained independently associated after multivariable adjustment [*β* = −1.26 [95% CI −2.06 to −0.46] for PCS; *β* = −1.50 [95% CI −2.40 to −0.61] for MCS; both *p* < 0.01]. Patients with moderate or higher depressive symptoms had significantly worse quality-of-life scores compared with those with mild symptoms. Mortality analyses were descriptive due to the limited number of events.

**Conclusions:**

In this cross-sectional study of STEMI survivors treated in a public hospital, depressive symptoms were highly prevalent at 30 days and were consistently associated with poorer physical and mental health-related quality of life. These findings highlight the substantial burden of depressive symptoms early after myocardial infarction and support the relevance of integrating mental health and quality-of-life assessment into post-infarction follow-up within public healthcare systems, while acknowledging the limitations of our small, single-centre study.

## Introduction

Cardiovascular diseases (CVD) are the leading cause of mortality worldwide and represent a substantial burden of morbidity (1). In 2021, CVD were estimated to be responsible for approximately 20 million deaths, accounting for nearly 30% of all global deaths, with projections suggesting a sustained increase in the coming decades. Within this group, ischemic heart disease stands out as the leading cause of premature death worldwide (2).

In Chile, age-adjusted cardiovascular mortality has declined steadily over recent decades; however, coronary heart disease continues to represent a major public health problem (3). Between 2000 and 2020, cardiovascular mortality decreased from 159.5 to 94.6 per 100,000 inhabitants, but mortality due to coronary causes reached approximately 41 deaths per 100,000 inhabitants in 2020, reflecting a persistent disease burden (4, 5).

Depression is frequently associated with cardiovascular disease and has been linked to worse clinical outcomes and higher mortality following coronary events. Population-based studies indicate that up to 20% of patients with coronary heart disease present concomitant anxiety–depressive symptoms (6). Beyond prevalence, the prognostic implications are substantial: depressive symptoms following acute myocardial infarction have been independently associated with a two- to threefold increase in all-cause and cardiovascular mortality, as well as with higher rates of rehospitalization, non-adherence to secondary prevention medications, and reduced engagement in cardiac rehabilitation (7). Moreover, the 2025 ESC Clinical Consensus Statement on mental health and cardiovascular disease recognises depressive symptomatology as a clinically relevant dimension of post-infarction care that warrants systematic evaluation.

In Chile, anxiety and depressive disorders represent a significant public health burden. According to the 2016–2017 National Health Survey, the 12-month prevalence of major depressive disorder was 6.2%, while the lifetime prevalence of depressive disorders reached 11.1% (8). While the relationship between depression and cardiovascular disease has been explored from pathophysiological perspectives, there is limited local clinical evidence characterizing, in a systematic manner, the burden of depressive symptoms and their relationship with quality of life in patients during the period following acute myocardial infarction, particularly from a public health perspective (9, 10).

Although the association between depressive symptoms and health-related quality of life has been extensively documented in international settings, local evidence characterising this relationship in public healthcare systems in Latin America (and specifically in Chile) remains scarce. This study contributes real-world data from a registry-based cohort of STEMI survivors treated exclusively in the public sector, a context associated with distinct socioeconomic and healthcare access constraints that may modify the burden and correlates of depressive symptoms. Furthermore, the use of both a validated clinician-rated instrument (HAM-D) and a widely used patient-reported outcome measure (SF-36) within the same prospective dataset allows an integrated characterisation of psychosocial burden that is seldom available in similar Latin American registries.

The primary objective of this study was to characterise health-related quality of life and the presence of depressive symptoms in patients following ST-segment elevation acute myocardial infarction. The secondary objective was to describe mortality patterns during the available follow-up in the study cohort, without formal association analyses given the limited number of events.

## Materials and methods

### Study design and participants

We conducted a cross-sectional observational study nested within the Prospective Myocardial Infarction Registry (REPRIM), which has been described in detail elsewhere (11). This registry prospectively enrolled patients with ST-segment elevation myocardial infarction (STEMI) treated at a single public tertiary care hospital in Los Angeles, Chile, between October 12nd 2019 and October 10th 2020.

During the study period, 121 patients with acute myocardial infarction were initially identified. Of these, 100 fulfilled the criteria for inclusion in the REPRIM registry, as reported in the original publication. For the present analysis, we focused on a predefined subcohort of patients with STEMI in whom health-related quality of life and depressive symptoms were assessed 30 days after the index event.

**Figure 1:**
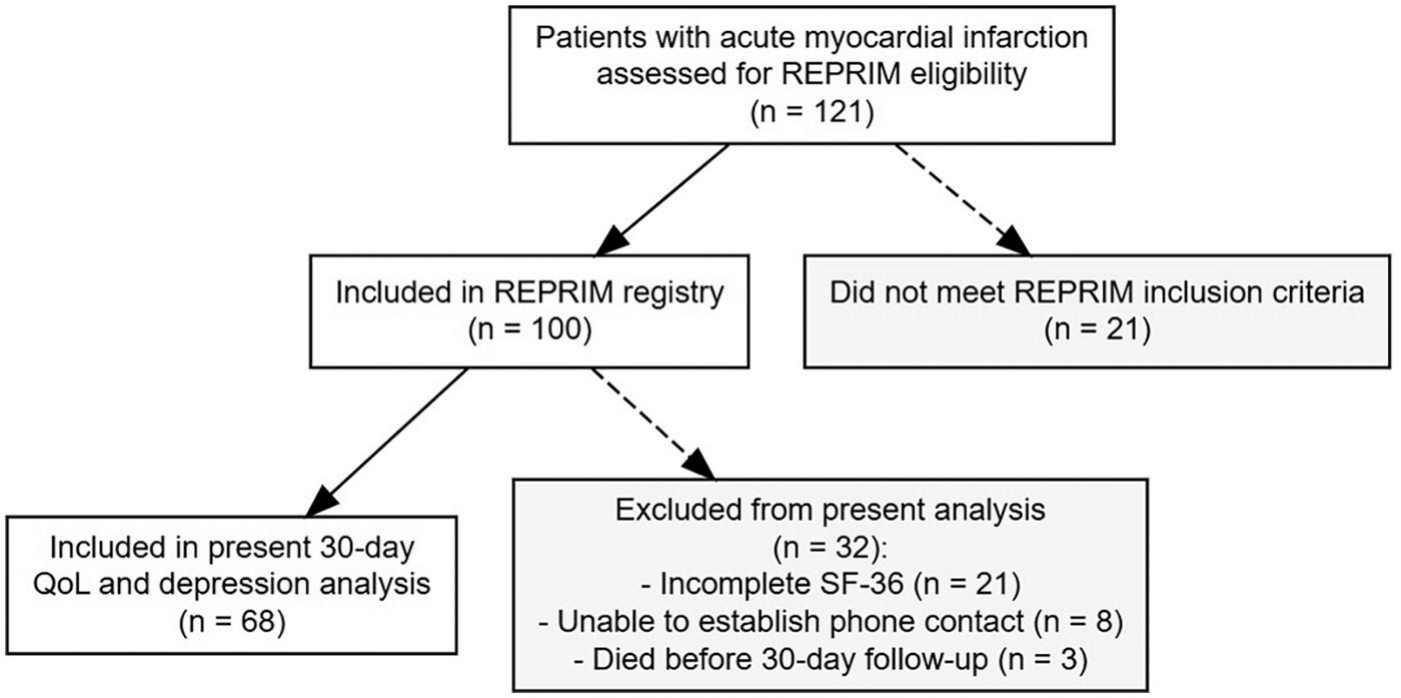
Patient flow for REPRIM registry and present sub-study.

From the 100 patients included in REPRIM, we included those who: (1) had a confirmed diagnosis of STEMI, (2) were alive at 30 days, (3) could be contacted for the 30-day follow-up interview, and (4) had complete data for the SF-36 and HAM-D scales, allowing computation of the summary scores. A total of 68 patients met these criteria and constituted the study population. The main reasons for non-inclusion in this subanalysis were death before the 30-day follow-up, inability to establish telephone contact, refusal to participate in the interview, and incomplete SF-36 or HAM-D questionnaires. A flow diagram summarizing patient selection, exclusions and reasons for non-participation is shown in Figure 1 in the Results section. All participants had provided written informed consent at the time of enrolment in the REPRIM registry. The study was conducted in accordance with the standards of the local scientific ethics committee and the principles of the Declaration of Helsinki.

### Assessment of health-related quality of life and depressive symptoms

Health-related quality of life was assessed at 30 days after the index STEMI using the SF-36 questionnaire. The eight standard domains (physical functioning, role physical, bodily pain, general health, vitality, social functioning, role emotional and mental health) were scored on a 0–100 scale, with higher scores indicating better perceived health status (12–14). For the present analysis, we used the Physical Component Summary (PCS) and Mental Component Summary (MCS) T-scores already derived from these domains in the study database, using established algorithms and normalized to a mean of 50 and standard deviation of 10 based on international reference values for ischemic heart disease (13, 15–17).

For the present analysis, we focused on PCS and MCS as summary measures because they provide a parsimonious and clinically interpretable characterisation of overall physical and mental health-related quality of life, reduce the multiple-comparison burden associated with analysing all eight domains individually, and are the most widely reported SF-36 outcomes in post-MI research, facilitating comparability with the existing literature (17). The eight individual domain scores are additionally presented in Figure 4 to allow a detailed inspection of the domain-level pattern across depressive symptom severity categories.

Depressive symptoms during the previous week were evaluated at the same 30-day follow-up using the 17-item Hamilton Depression Rating Scale (HAM-D). The total HAM-D score was obtained by summing all items, with higher scores reflecting greater depressive symptom severity (17, 18). In accordance with widely used thresholds, we categorized HAM-D scores as 0–7 (no depression/remission), 8–16 (mild depression), 17–23 (moderate depression) and ≥24 (severe depression) (18–20). In addition, for some comparative analyses we grouped patients into “mild” (HAM-D 8–13) vs. “moderate or higher” (HAM-D ≥ 14), reflecting the score distribution in our cohort and the limited sample size; this analytical grouping is explicitly reported in the Results.

The 30-day timepoint was selected because it represents an early and clinically meaningful phase of post-infarction recovery, when initial physical limitations, psychological responses and treatment adherence patterns are being established, and often coincides with the first structured follow-up contact in our setting. We acknowledge that depressive symptoms and quality of life may still be evolving at this stage, which is discussed as a limitation.

### Clinical variables and follow-up

All clinical and outcome variables used in this study were obtained from the curated analysis dataset derived from REPRIM. The following variables were available and used in the analyses: vital status (alive/deceased), date of death, and derived survival time in days, months and years from the index myocardial infarction; age; sex; rural vs. urban residence; history of diabetes mellitus and hypertension; active smoking status; high-density and low-density lipoprotein cholesterol (HDL, LDL); serum creatinine; use at 30 days of dual antiplatelet therapy, beta-blockers, renin–angiotensin system inhibitors, spironolactone and loop diuretics; left ventricular ejection fraction (LVEF) measured by the biplane method; HAM-D total score and categorical severity; and SF-36 domain scores and PCS/MCS T-scores.

Vital status was ascertained through linkage with the Chilean Civil Registry and Identification Service and updated up to 31 December 2025. Mortality was summarised descriptively at 30 days, 1 year and 5 years after STEMI, and overall follow-up time was described using median and interquartile range. Given the limited number of events, we did not perform formal association analyses between PCS/MCS or HAM-D and mortality; this is addressed in the Results and Limitations.

### Statistical analysis

Continuous variables were assessed for normality using the Shapiro–Wilk test. In the study sample, none of the continuous variables (including HAM-D, PCS, MCS, age and LVEF) passed the Shapiro–Wilk normality criterion at the conventional *p* > 0.05 threshold, supporting the use of non-parametric tests for most bivariable comparisons. The two-sample comparisons for PCS and MCS by depressive severity group were therefore performed using the Mann–Whitney *U*-test, and comparisons across more than two groups were performed using the Kruskal–Wallis test. Results are presented as means with standard deviations or medians with interquartile ranges, as appropriate. For multivariable linear regression models, the assumptions of linearity, homoscedasticity and absence of influential outliers were visually inspected using residual plots; no major violations were identified.

The multivariable models covariates were selected *a priori* based on their clinical relevance and their established associations with both depressive symptomatology and health-related quality of life in post-MI populations, as documented in the prior literature (7, 21, 22). Age and sex are demographic determinants of both SF-36 scores and depressive burden; diabetes mellitus and hypertension represent comorbidity burden that may independently affect both outcomes; and LVEF captures the severity of myocardial injury, which may influence physical functioning and psychological adaptation. Although other potential confounders (e.g., socioeconomic status, antidepressant use, rehabilitation participation) are conceptually plausible, their systematic absence from the dataset precluded their inclusion. We acknowledge this as a limitation and note that residual confounding cannot be excluded.

Given the small sample size, formal tests of regression assumptions are reported with caution and results should be interpreted accordingly.

All analyses for this manuscript were performed using RStudio (version 2026.01.1, Build 403).

## Results

### Study population

Of the 121 patients with acute myocardial infarction identified during the study period, 100 were included in the REPRIM registry. Among these, 68 patients with STEMI met the predefined criteria for the present subanalysis (alive at 30 days, successful contact for the follow-up interview and complete SF-36 and HAM-D data) and constituted the study population. The main reasons for non-inclusion in this analysis were death before the 30-day follow-up, inability to establish telephone contact, refusal to participate in the interview and incomplete questionnaire data. A flow diagram describing patient selection, exclusions and reasons for non-participation is shown in Figure 1.

Baseline characteristics of the 68 included patients are summarised in Table 1. The median age was 60.5 years [interquartile range (IQR) 53.8–70.0], and 76.5% were male. Hypertension and diabetes mellitus were present in 64.7% and 27.9% of patients, respectively, and 41.2% were active smokers. The median LVEF was 46.0% (IQR 40.8–54.2) among patients with available echocardiographic data. Compared with the remaining patients in the REPRIM registry, individuals included in this subanalysis showed similar distributions of age, sex, diabetes, hypertension and LVEF, suggesting no major differences in these key baseline characteristics, although selection and survivorship bias cannot be ruled out.

**Table 1:** Cohort characteristics.

Variable	Total (*n* = 68)
Age, years	60.5 (53.8–70.0)
Male sex, *n* (%)	52 (76.5)
Rural residence, *n* (%)	23 (33.8)
Diabetes mellitus, *n* (%)	19 (27.9)
Hypertension, *n* (%)	44 (64.7)
Active smoking, *n* (%)	28 (41.2)
HDL cholesterol (mg/dL)	36.5 (30.8–42.2)
LDL cholesterol (mg/dL)	110.5 (89.0–137.2)
Serum creatinine (mg/dL)	0.9 (0.8–1.2)
Dual antiplatelet therapy, *n* (%)	63 (92.6)
Beta-blocker therapy, *n* (%)	49 (72.1)
ACE inhibitor therapy, *n* (%)	45 (66.2)
Spironolactone, *n* (%)	9 (13.2)
Diuretic therapy (furosemide), *n* (%)	8 (11.8)
Biplane left ventricular ejection fraction (%)*	46.0 (40.8–54.2)
HAM-D score	12.0 (10.0–15.0)
PCS (T-score)	45.6 ± 9.2
MCS (T-score)	49.0 ± 10.3

Continuous variables are presented as median (interquartile range) or mean  ± standard deviation, according to data distribution. Categorical variables are expressed as *n* (%). PCS and MCS are reported as T-scores (reference population mean = 50, SD = 10). *Left ventricular ejection fraction (LVEF) was available in a subset of patients.

### Depressive symptoms at 30 days

At 30 days after STEMI, the median HAM-D score was 12 points (IQR 10–15), with values ranging from 1 to 23. Overall, 4 patients (5.9%) had HAM-D scores <7, consistent with no depression or remission, whereas 40 (58.8%) were classified as having mild depressive symptoms, 23 (33.8%) as moderate and 1 (1.5%) as severe according to standard cut-points. Thus, a large majority of the cohort presented at least mild depressive symptoms, with most patients concentrated in the mild and moderate categories.

For comparative analyses, patients were additionally grouped into “mild” (HAM-D 8–13) and “moderate or higher” (HAM-D ≥ 14). Using this analytical categorisation, 41 patients (60.3%) fell into the mild group and 22 (32.4%) into the moderate-or-higher group, while the small number of patients with HAM-D < 7 was described separately.

### Health-related quality of life

Health-related quality of life at 30 days is summarised in Table 1. The mean PCS T-score was 45.6 ± 9.2 and the mean MCS T-score was 49.0 ± 10.3, both slightly below the reference mean of 50 for the general population. The eight SF-36 domains showed heterogeneous patterns, with lower scores observed particularly in domains related to physical functioning and vitality, while social functioning and role emotional were relatively preserved in many patients.

In univariate analyses, there was no clear linear association between age (as a continuous variable) and PCS or MCS. However, when age was categorised into quartiles, PCS differed significantly across age groups (Kruskal–Wallis *H* = 8.40; *p* = 0.038), whereas MCS did not show significant differences. No statistically significant differences in PCS, MCS or HAM-D scores were observed across subgroups defined by sex, diabetes, hypertension, rural vs. urban residence, smoking status or pharmacological treatments at 30 days.

### Association between quality of life and depressive symptoms

**Figure 2:**
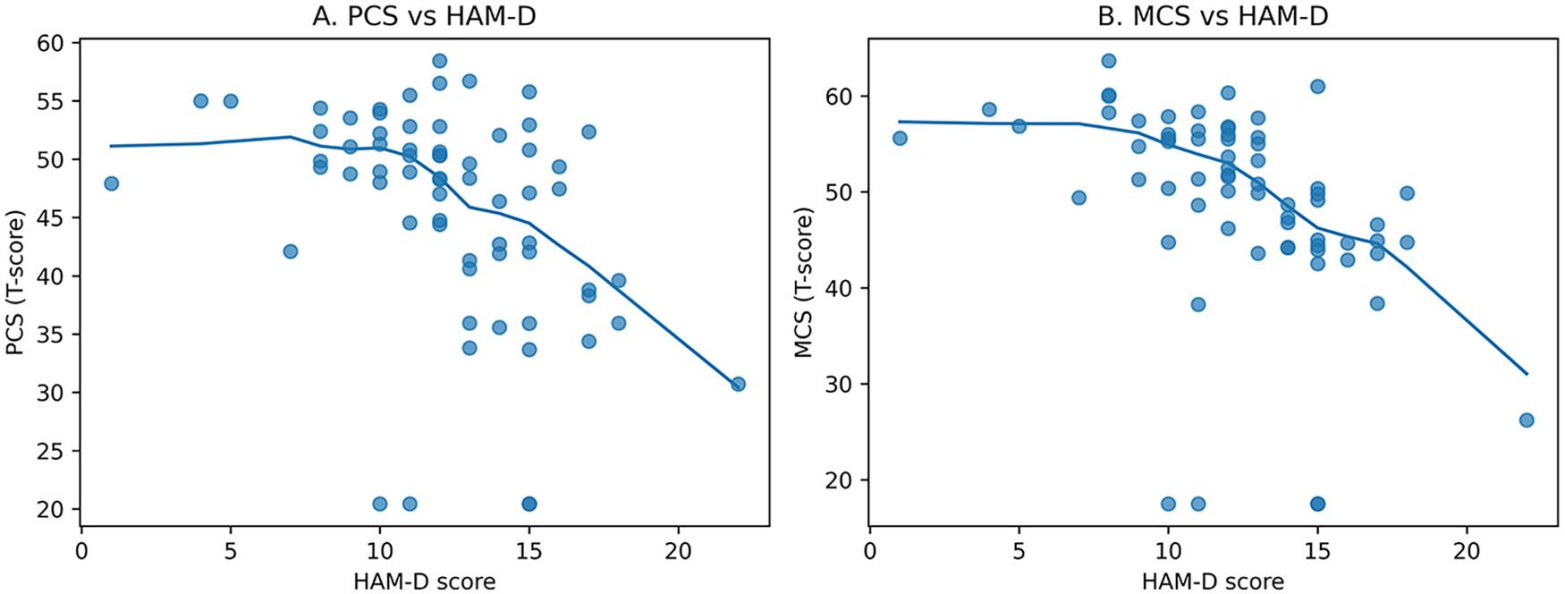
Scatter plots showing the association between Hamilton depression rating scale (HAM-D) scores and the physical (PCS) and mental (MCS) components of the SF-36 questionnaire. Panel A shows PCS vs. HAM-D, and Panel B shows MCS vs. HAM-D. The solid line represents a non-parametric smoothing function. A significant inverse association was observed between depressive symptoms and both quality-of-life components (*p* < 0.001).

A significant inverse association was observed between HAM-D scores and both SF-36 summary components. Higher depressive symptom severity correlated with lower PCS (Spearman's *ρ* = −0.46; *p* < 0.001) and lower MCS (*ρ* = −0.58; *p* < 0.001), with a stronger correlation for the mental component. These relationships are illustrated in Figure 2.

**Figure 3:**
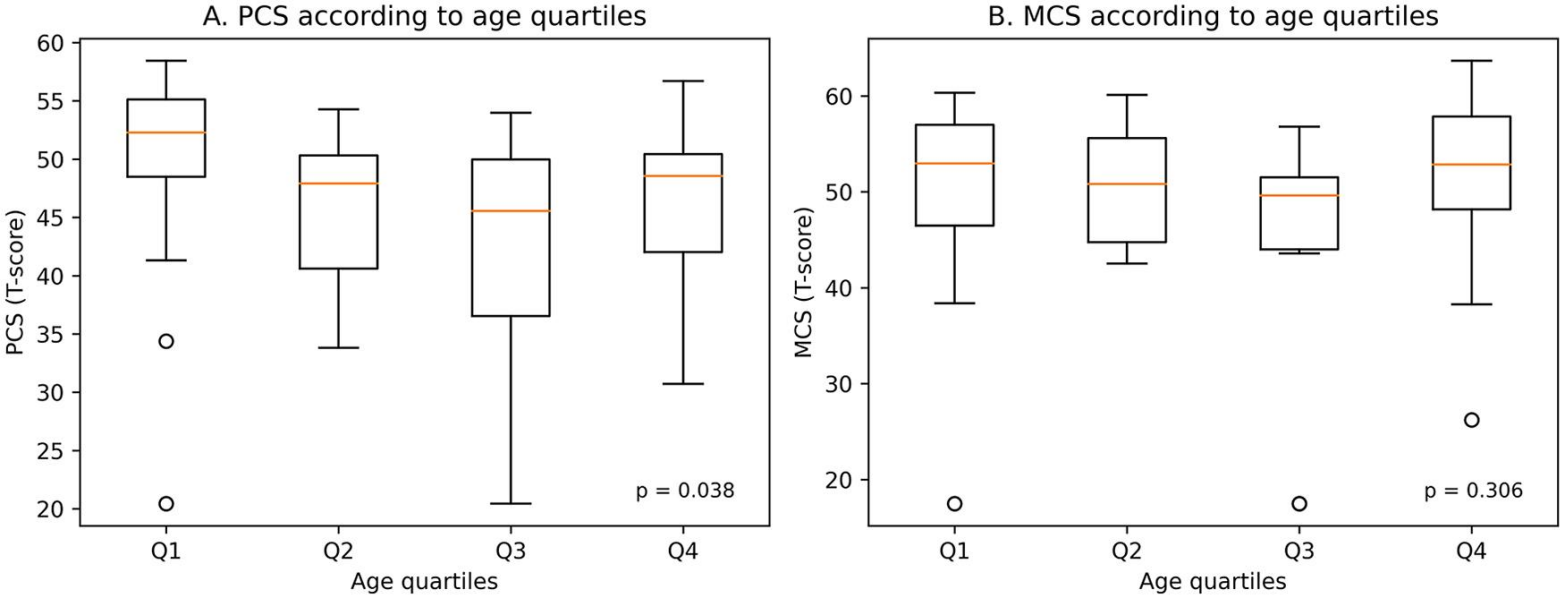
Box plots of physical component summary (PCS, panel **A**) and mental component summary (MCS, panel **B**) of the SF-36 according to depressive symptom severity (HAM-D 8–13 vs. ≥14). Boxes represent the median and interquartile range; whiskers indicate 1.5 × IQR. Group differences were assessed using the Mann–Whitney *U*-test (*p* < 0.01 for PCS; *p* < 0.001 for MCS).

**Figure 4:**
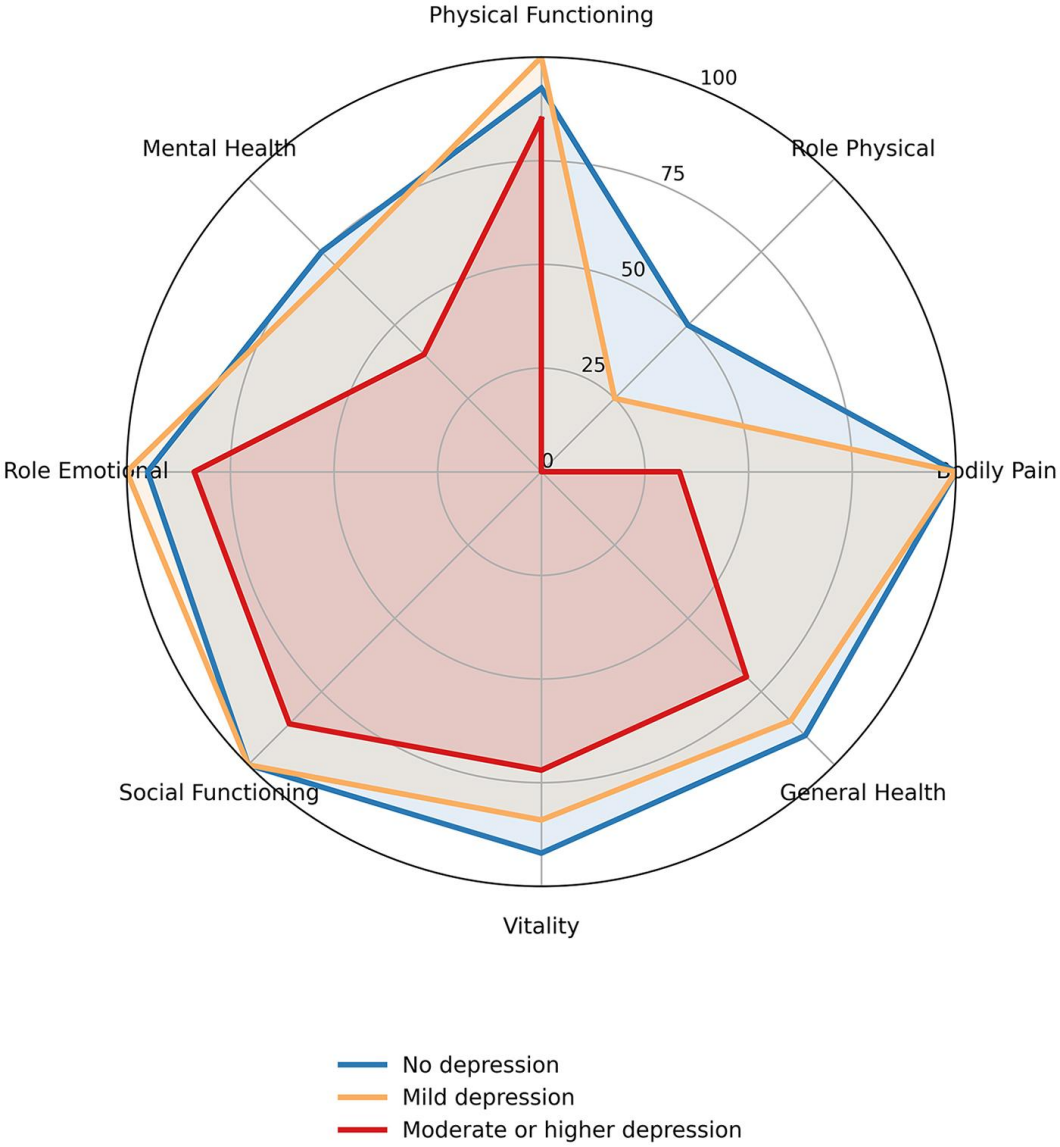
Radar plot comparing the eight SF-36 domains across depressive symptom severity categories assessed by the HAM-D: no depression, mild depression, and moderate or higher depression. Values correspond to the median score of each domain.

When patients were grouped by depressive symptom severity, those with moderate or higher depression (HAM-D ≥ 14) had markedly worse quality-of-life scores compared with those with mild symptoms (HAM-D 8–13). Median PCS values were lower in the moderate-or-higher group than in the mild group (42.4 vs. 50.3; Mann–Whitney *U*, *p* < 0.001), and the difference was even more pronounced for MCS (44.7 vs. 55.5; *p* < 0.001), as shown in Figure 3. Radar plots of the eight SF-36 domains (Figure 4) highlight a consistently poorer profile across all domains with increasing depressive symptom severity, confirming that the burden of symptoms extends beyond purely mental health dimensions.

### Multivariable analyses

In multivariable linear regression analyses, higher depressive symptom severity remained independently associated with poorer health-related quality of life after adjustment for age, sex, diabetes mellitus, hypertension and LVEF. For the physical component of the SF-36 (PCS), each 1-point increase in HAM-D score was associated with a 1.26-point lower PCS value (*β* = −1.26; 95% CI −2.06 to −0.46; *p* = 0.003). For the mental component (MCS), each 1-point increase in HAM-D score was associated with a 1.50-point lower MCS value (*β* = −1.50; 95% CI −2.40 to −0.61; *p* = 0.002). These findings indicate that the inverse relationship between depressive symptoms and both physical and mental aspects of quality of life is robust to adjustment for key clinical covariates, although residual confounding cannot be excluded.

### Mortality

**Figure 5:**
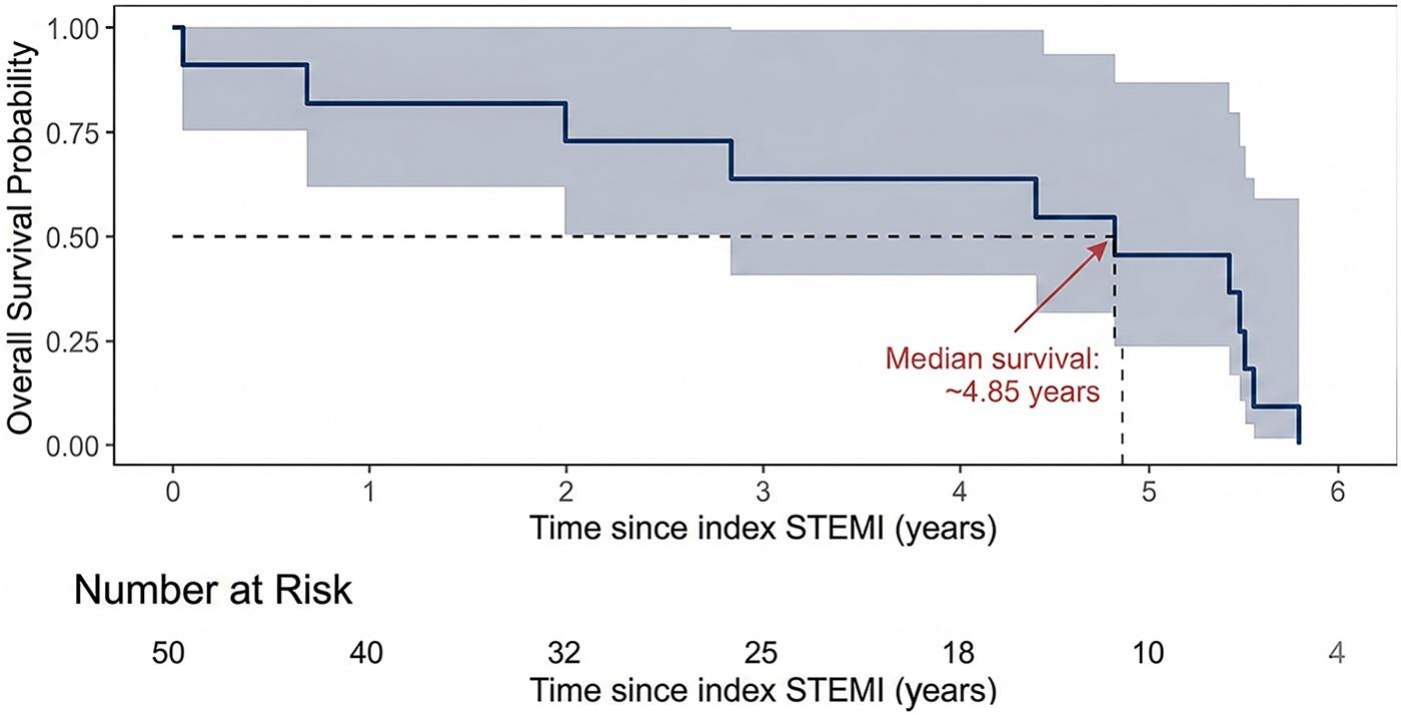
Kaplan–meier curve for overall survival after STEMI throughout the available follow-up. The solid blue line represents the Kaplan–Meier survival estimate, and the shaded area indicates the 95% confidence interval. Vertical tick marks denote censored observations (patients alive at last follow-up).

During follow-up, 30-day mortality after STEMI was 4.4% (3 deaths), cumulative 1-year mortality was 11.8% (8 deaths) and 5-year mortality was 16.2% (11 deaths). A Kaplan–Meier survival curve is presented in Figure 5, illustrating the stepwise decline in overall survival probability over the follow-up period, with relatively few deaths occurring after the first year. The number of patients at risk at each time point is shown in the accompanying risk table. Given the low number of events, mortality analyses were considered descriptive and exploratory, and no formal association analyses between PCS/MCS or HAM-D and mortality were performed.

## Discussion

In this cross-sectional analysis of a registry-based cohort of STEMI survivors treated in a public healthcare setting, we observed a high burden of depressive symptoms at 30 days and a consistent inverse association between depressive symptom severity and health-related quality of life. Most patients presented at least mild depressive symptoms, and higher HAM-D scores were associated with worse SF-36 physical and mental component scores, both in unadjusted analyses and after adjustment for key clinical covariates. These findings highlight the close interplay between mental health and perceived health status early after myocardial infarction and underscore the relevance of integrating psychosocial dimensions into secondary prevention strategies.

### Prevalence of depressive symptoms

The very high proportion of patients with at least mild depressive symptoms in our cohort contrasts with the prevalence of major depression usually reported in post-MI populations, which is often in the range of 20%–30% (7, 21, 22). This comparison must be interpreted with caution: the figure of 20%–30% typically refers to clinically significant depressive episodes assessed through structured diagnostic interviews (e.g., DSM or ICD criteria) or validated cut-offs on instruments such as the PHQ-9 or BDI, whereas our study used the HAM-D to characterise depressive symptom severity on a continuum. The HAM-D captures a broader spectrum of symptoms, and mild elevations above commonly used thresholds may not correspond to syndromic major depressive disorder.

Taken together, these considerations suggest that our estimates are best interpreted as reflecting the burden of depressive symptoms in a vulnerable post-STEMI population rather than as precise prevalence figures for clinically diagnosed depressive disorders. Nonetheless, the high symptom burden is clinically meaningful, as even mild depressive symptoms have been associated with poorer adherence, functional limitations and reduced quality of life in cardiovascular populations (7, 21, 22).

### Health-related quality of life and reference values

The mean PCS and MCS T-scores at 30 days were slightly below the reference mean of 50, consistent with impaired health-related quality of life in this setting and in line with international reference values for patients with ischemic heart disease (13, 15–17). The pattern across SF-36 domains, with lower scores particularly in physical functioning and vitality, is compatible with the expected impact of STEMI on early physical recovery and energy levels. Radar plots showed a progressive deterioration in all domains with increasing depressive symptom severity, suggesting that depression-related distress may permeate both physical and psychosocial aspects of daily life rather than being confined to mental health constructs alone.

It should be noted, however, that the absence of pre-MI baseline data on quality of life or depressive symptoms in our cohort precludes a direct comparison with pre-STEMI levels and limits the ability to attribute observed impairments exclusively to the myocardial infarction event. This is a key limitation that affects the interpretation of the clinical relevance of our estimates.

### Association between depressive symptoms and quality of life

The present analysis examined depressive symptom severity as the primary independent variable and health-related quality of life as the outcome, following the convention of much of the published literature in this field (7, 21, 22). However, we acknowledge that this directional assumption is not empirically justified within our cross-sectional design. A plausible alternative framework posits that impaired physical functioning and perceived health status (as captured by SF-36 PCS), may themselves precipitate or amplify depressive reactions, particularly in the early post-infarction period when patients face abrupt limitations in daily activities and increased uncertainty about prognosis. The biological and psychological mechanisms supporting this bidirectional relationship (e.g., activation of the hypothalamic–pituitary–adrenal axis, inflammatory pathways, reduced self-efficacy) have been extensively reviewed elsewhere (7). In the absence of longitudinal data with temporally ordered measurements, we are unable to disentangle the direction of the association, and the findings should be interpreted as reflecting a co-occurrence of depressive symptoms and reduced quality of life rather than a directional causal relationship.

As anticipated, the strongest crude correlation was observed between HAM-D and the SF-36 mental component, given the conceptual overlap between depressive symptoms and the MCS domains (emotional well-being, vitality, social functioning). However, the association with the physical component is particularly informative: higher HAM-D scores were associated not only with worse MCS, but also with lower PCS, even after adjustment for age, sex, diabetes, hypertension and LVEF. Each additional point in HAM-D was associated with a 1.26-point lower PCS and a 1.50-point lower MCS, with confidence intervals clearly excluding the null. This pattern supports the notion that depressive symptoms and physical recovery after STEMI are closely intertwined and that mental health may influence patients perception of physical functioning, symptom burden and overall capacity to engage in rehabilitation.

From a practical standpoint, SF-36 and HAM-D provide complementary information. The SF-36 offers a broad, patient-reported profile of health-related quality of life, capturing both physical and psychosocial domains, while HAM-D provides a clinician-rated, granular assessment of depressive symptom severity that may better inform diagnostic and therapeutic decisions in mental health. In resource-limited settings, SF-36 MCS could be used as a pragmatic screening tool to identify patients with impaired mental health-related quality of life, whereas HAM-D might be reserved for cases requiring more detailed assessment or psychiatric referral. Our results support the combined use of these instruments in post-STEMI care pathways, particularly where structured cardiac rehabilitation and psychosocial support are available (7).

Whether the observed association between depressive symptoms and health-related quality of life differs in magnitude or pattern from that reported in post-MI populations in other countries or healthcare settings remains an open question. Our study was not designed to test this hypothesis formally, but the consistency of the direction of association with international evidence is reassuring. Future comparative studies with matched general population data would help contextualise these findings.

### Context of public healthcare and implications for care

Our study was conducted in a public tertiary care context in Chile, contributing data from a healthcare setting and region that are underrepresented in the literature on post-MI depression and quality of life. The high burden of depressive symptoms and reduced quality of life observed in this sample aligns with recent statements from international societies, such as the American Heart Association and the European Society of Cardiology, which emphasise the importance of identifying and addressing psychological factors in cardiovascular care (7, 21). In Chile, the National Cardiovascular Health Program has incorporated depression screening for patients with chronic diseases, and national technical documents recognise cardiac rehabilitation as a key setting to address both physical and psychosocial dimensions of cardiovascular care (23).

Within this framework, our findings reinforce the relevance of systematically considering mental health and quality of life in post-infarction follow-up, particularly in public healthcare systems where structural and resource constraints may limit access to specialised psychosocial services. Even though our study cannot establish causality or evaluate the impact of specific interventions, the observed associations support the rationale for incorporating depression and quality-of-life assessments into routine post-STEMI evaluations and for integrating psychosocial components into rehabilitation programmes and long-term secondary prevention strategies (7, 21).

### Limitations

This study has several limitations that should be acknowledged. First, the cross-sectional design and the absence of pre-MI or admission data on depression and quality of life prevent us from determining whether the observed symptoms are new-onset or pre-existing and preclude any inference about causal or temporal relationships between depressive symptoms, quality of life and clinical outcomes (7, 21, 22). The inability to distinguish incident from pre-existing depressive symptoms is a substantive limitation: patients with pre-existing depression may have had impaired quality of life prior to their myocardial infarction, in which case the observed associations would reflect, at least in part, a pre-existing psychological vulnerability rather than a consequence of the cardiac event. This precludes any conclusion about the directional or causal nature of the findings. Furthermore, the cross-sectional design inherently precludes causal inference: both the co-occurrence of depressive symptoms and impaired quality of life may be driven by a third common pathway (e.g., severity of the infarction, pain, functional limitation), and the possibility of reverse causality (whereby reduced physical or mental quality of life generates or amplifies depressive reactions) cannot be excluded. These conceptual limitations are discussed in the context of bidirectionality in the Association section above.

Second, the effective sample size of 68 patients, and particularly the number of events for mortality analyses, is modest and limits statistical power, especially for subgroup comparisons and multivariable models. Although the patients included in this subanalysis were broadly similar to the remainder of the REPRIM cohort in key baseline characteristics, selection and survivorship bias are possible, as only survivors who could be contacted and agreed to complete the 30-day interview were included.

Third, detailed information on mental health treatment, psychotherapy, antidepressant use and participation in cardiac rehabilitation was not systematically available in the present dataset, which limits our ability to interpret symptom burden in light of concurrent psychosocial or behavioural interventions.

Fourth, the multivariable linear regression models for PCS and MCS included a restricted set of covariates, selected *a priori* for clinical relevance, to avoid overfitting given the limited number of observations; residual confounding by unmeasured or incompletely measured variables cannot be excluded.

Fifth, mortality analyses were largely descriptive, and exploratory Cox models with PCS or MCS as predictors did not yield statistically robust associations with survival, with wide confidence intervals reflecting the small number of deaths. Accordingly, we refrained from presenting detailed survival models and limited our conclusions to descriptive mortality patterns.

Sixth, no formal power calculation was performed for this subanalysis, as the sample corresponds to all eligible patients from the registry during the study period. A *post-hoc* sensitivity analysis indicated that, for a Spearman correlation of *ρ* = −0.46 between HAM-D and PCS (the weakest of the two main associations), a sample of 68 provided approximately 99% power to detect this correlation at α = 0.05 (two-sided), suggesting that the primary correlation analyses were adequately powered. However, the multivariable regression models and subgroup comparisons operated with substantially reduced degrees of freedom, limiting the precision of those estimates and their generalisability to the broader target population.

Finally, although normality was assessed for all continuous variables using the Shapiro–Wilk test and test selection was guided by these results, formal verification of all regression assumptions (e.g., independence of residuals, absence of multicollinearity) was limited by the small sample size, and some diagnostic procedures may have had reduced sensitivity in this context.

Despite these limitations, our study has several strengths, including the use of validated instruments (SF-36 and HAM-D), the focus on a real-world STEMI cohort in a public healthcare setting and the availability of long-term mortality data. Together, these features provide a useful, albeit preliminary, contribution to understanding the burden and correlates of depressive symptoms and impaired quality of life in post-MI patients in a Latin American context.

## Conclusion

This study demonstrates that depressive symptoms are highly prevalent in patients evaluated after acute myocardial infarction and are consistently associated with poorer health-related quality of life, even after adjustment for key clinical covariates.

These findings support the importance of systematically incorporating mental health and quality-of-life assessments into post-infarction follow-up and reinforce the need for future prospective studies and multidisciplinary interventions in this field, particularly within public healthcare systems.

## Data Availability

The datasets presented in this article are not readily available because The data collected and generated are protected under Chilean legislation regarding personal data protection, research involving human subjects, and patients' rights and duties; therefore, they cannot be shared with third parties. Requests to access the datasets should be directed to manuel.mallol@gmail.com.
